# Supplementary material for: A strategy to detect metabolic changes induced by exposure to chemicals from large sets of condition-specific metabolic models computed with enumeration techniques
Source: BMC Bioinformatics. 2024 Jul 11;25:234. doi: 10.1186/s12859-024-05845-z (PMC11238488; doi:10.1186/s12859-024-05845-z)
Supplement: Supplementary file 1 — Supplementary Material 1: S1 Appendix. Supplementary information. Pdf including: Correction coefficient formula, DAR specificity ratio formula, DEXOM parameters, systematic sampling and robustness analysis. [file 12859_2024_5845_MOESM1_ESM.pdf]

A strategy to detect metabolic changes induced by exposure to chemicals from large sets of condition-specific metabolic models computed with enumeration techniques

Louison Fresnais[1,2]*, Olivier Perin[2], Anne Riu[2], Romain Grall[2], Alban Ott[2], Bernard Fromenty[3], Jean-Clément Gallardo[1], Maximilian Stingl[1], Clément Frainay[1], Fabien Jourdan[1,4] and Nathalie Poupin[1]*

1 UMR1331 Toxalim (Research Centre in Food Toxicology), Université de Toulouse, INRAE, ENVT, INP-Purpan, UPS, Toulouse, France.

2 L'Oréal Research and Innovation, Aulnay-sous-Bois, France.

3 INSERM, Univ Rennes, INRAE, Institut NUMECAN (Nutrition Metabolisms and Cancer) UMR_A 1317, UMR_S 1241, F-35000 Rennes, France

4 MetaboHUB-MetaToul, National Infrastructure of Metabolomics and Fluxomics, Toulouse, France

*To whom correspondence should be addressed.

Supplementary

Interactive visualizations with MetExploreViz:

Fig 3A:

https://metexplore.toulouse.inrae.fr/userFiles/metExploreViz/index.html?dir=/5b6c886c4916c1de9e6c16a776cc6d64/networkSaved_1726726315

Fig 3B:

https://metexplore.toulouse.inrae.fr/userFiles/metExploreViz/index.html?dir=/5b6c886c4916c1de9e6c16a776cc6d64/networkSaved_1522683843

Fig 5A:

https://metexplore.toulouse.inrae.fr/userFiles/metExploreViz/index.html?dir=/5b6c886c4916c1de9e6c16a776cc6d64/networkSaved_292937465

Fig 5B:

https://metexplore.toulouse.inrae.fr/userFiles/metExploreViz/index.html?dir=/5b6c886c4916c1de9e6c16a776cc6d64/networkSaved_1994092833

Fig 6A:

https://metexplore.toulouse.inrae.fr/userFiles/metExploreViz/index.html?dir=/5b6c886c4916c1de9e6c16a776cc6d64/networkSaved_373423088

Fig 6B:

https://metexplore.toulouse.inrae.fr/userFiles/metExploreViz/index.html?dir=/5b6c886c4916c1de9e6c16a776cc6d64/networkSaved_725935955

S4 FigA:

https://metexplore.toulouse.inrae.fr/userFiles/metExploreViz/index.html?dir=/5b6c886c4916c1de9e6c16a776cc6d64/networkSaved_682018369

S4 FigB:

https://metexplore.toulouse.inrae.fr/userFiles/metExploreViz/index.html?dir=/5b6c886c4916c1de9e6c16a776cc6d64/networkSaved_1293062392

Correction coefficient:

To maintain the biomass reaction balanced after setting the stoichiometry coefficient for the biomass_DNA reactant to zero, we applied the following correction coefficient to the stochiometric coefficient for all other reactants of the biomass reaction:

$$corCoef = \frac{1}{1 - S_{DNA}}$$

Where S_{DNA} refers to the stoichiometric coefficient for the biomass_DNA reactant in the biomass reaction initially defined in Recon2.2 [1]. We applied this correction coefficient to all other metabolites (i.e biomass_lipid, biomass_carbohydrate, biomass_other, biomass_RNA and biomass_protein) of the Recon2.2 biomass reaction so that the sum of the coefficients remains equal to one.

DAR specificity ratio:

To evaluate the degree of specificity of a Differentially Activated Reaction (DAR) signature, we compute the DAR specificity ratio. A DAR specificity ratio of 100% means that all the predicted DARs are retrieved only for the studied molecule, meaning that the metabolic Mechanism of Action (mMoA) of this molecule is unique compared to mMoA of other studied molecules.

$$DARspec_x = \frac{nSpecificDARs_x}{nTotalDARs_x}$$

With $nSpecificDARs_x$, the number of DARs retrieved only in the list of DARs predicted for the molecule X and $nTotalDARs_x$, the total number of DARs predicted for the molecule X.

DEXOM parameters:

The choice of parameters values is based on a compromise between the quality of returned solutions and the solving time.
Indeed, we can compare the optimality score achieved by the algorithm to the theoretical optimality score of the constructed mixed integer linear problem which is the maximum adequacy score between the network topology of the GSMN and the list of active/inactive reactions obtained from the binarization of transcriptomic data.
We selected the following parameters:

- eps = $1e^{-2}$
- thr = $1e^{-5}$
- tlim = 600s
- mipgaptol = $1e^{-3}$

eps and thr are the thresholds above which a reaction is considered active. eps represent, the activation threshold of activated reactions (according to transcriptomic data) and thr represents, the activation threshold for unweighted reactions (according to transcriptomic data). tlim is the parameter defining the maximum time allowed to solve a mixed integer linear problem. The mipgaptol parameter defines the maximal allowed gap between the theoretical maximal adequacy score and the adequacy score of the optimized solution allowing us to consider the solution as optimal. The trade-off between quality and running time described above is mainly affected by tlim and mipgaptol. Therefore, these two parameters might need to be carefully adjusted according to the complexity of the problem and the available computational resources.

Adapted version of Systematic sampling:

Systematic sampling aims at picking random samples in a uniform fashion over all the dataset [2]. Here, the dataset consists of solutions enumerated with the Reaction-Enum partial enumeration method. We implemented this systematic sampling method because in Recon2.2, reactions are sorted in alphabetical order by default and some reactions names are informative of their function (*e.g.* EX_R… refers to Exchange reactions). Since the aim of Reaction-Enum is to iteratively block each reaction and if possible, find an optimal solution with this reaction blocked, the order of solutions returned by Reaction-Enum is impacted by this reaction naming bias.

Therefore, to avoid picking an imbalanced set of starting solutions (*i.e.* a set of solutions not representative of the diversity of possible solutions computed by Reaction-Enum), we implemented a systematic sampling approach.

First, we compute the number of batches according to the size of the range defined at the beginning of the batch computation of the Reaction-Enum step. Then for each batch we pick a random solution in the corresponding range of Reaction-Enum solutions. This solution will then be used as starting point of a Diversity-Enum batch. As an example, in the case where we have 4500 solutions obtained by Reaction-Enum and a range of 100 solutions per batch, the starting solution for the first Diversity-Enum batch will be picked between the first and 99th Reaction-Enum solutions, the starting solution for the second Diversity-Enum batch will be picked between the 100th and the 199th Reaction-Enum solutions and so on until we picked a starting solution for each batch, the last one being between the 4400th and the 4500th Reaction-Enum solutions

Impact of DARs filtration on pathway enrichment results:

Pathway over-representation analysis (ORA) is size sensitive [3,4]. Therefore, keeping reactions that are not functionally informative (*i.e.* sink, pool and exchange reactions) can lead to important differences in pathway ORA. For instance, when keeping such reactions, only one significantly enriched pathway (Transport, extracellular) was evidenced for valproic acid (S3 Fig), whereas 12 were identified when removing these reactions. These results suggest that DARs lists should be carefully processed in order to remove such reactions before performing an over-representation analysis. Interestingly, the graph-based analysis developed in this study is less impacted by keeping or removing these reactions since they are usually clustered together.

Robustness of the results to the random-selection of starting solutions

Due to the high computation cost of DEXOM, we adapted the sampling strategy by using only 1% of the solutions enumerated by the reaction-enum approach as starting solutions for the diversity-enum approach. To assess the robustness of the results after this adaptation we performed 5 runs of our approach, by randomly selecting a different set of 1% reactions in each run. This test was done on two conditions: amiodarone, control, 24hr and amiodarone, high dose, 24hr.

We first checked that the overlap between randomly selected sets across the 5 runs was low, so that we would be able to truly assess the impact of the stochasticity of the random selection process on the robustness and consistency of our results.
Table 1 and Table 2 show that the overlap of randomly selected reaction-enum solutions is always lower than 5%, highlighting that starting solutions for each of the 5 runs are indeed very different.

	run1	run2	run3	run4	run5
run1		4.07%	2.46%	1.64%	1.64%
run2			1.63%	4.07%	3.25%

run3				2.46%	3.28%
run4					3.28%
run5					

Table 1. Overlap between the solutions from reaction-enum selected in each run for the amiodarone control condition at 24hr.

	run1	run2	run3	run4	run5
run1		1.61%	1.61%	2.42%	4.03%
run2			2.42%	1.61%	3.23%
run3				3.23%	2.42%
run4					1.61%
run5					

Table 2. Overlap between the solutions from reaction-enum selected in each run for the amiodarone high dose condition at 24hr.

We next compared the overlap between DARs calculated from each run. The results (Table 3) show that the solutions enumerated by the diversity-enumeration approach are very similar across the 5 runs, with a lowest overlap of 97.2%, which corresponds to only 1 DAR being different between the 2 runs.
These simulations show that our adaptation of DEXOM does not significantly impact the diversity of the enumerated solution, and we therefore consider that this adaptation is relevant to allow a drastic reduction in computing time while providing robust and consistent results.

	run1	run2	run3	run4	run5
run1		97.18%	100.00%	100.00%	100.00%
run2			97.18%	97.18%	97.18%
run3				100.00%	100.00%
run4					100.00%
run5					

Table 3. Overlap between predicted DARs in each run for the amiodarone condition.

References:

1. Swainston N, Smallbone K, Hefzi H, Dobson PD, Brewer J, Hanscho M, et al. Recon 2.2: from reconstruction to model of human metabolism. Metabolomics. 2016;12:109.

2. A S, A I. Recent developments in systematic sampling: A review. J Stat Theory Pract [Internet]. 2018;12(2):290–310. Available from: https://doi.org/10.1080/15598608.2017.1353456

3. Karp PD, Midford PE, Caspi R, Khodursky A. Pathway size matters: the influence of pathway granularity on over-representation (enrichment analysis) statistics. BMC Genomics [Internet]. 2021;22(1):191. Available from: https://doi.org/10.1186/s12864-021-07502-8

4. Wieder C, Frainay C, Poupin N, Rodríguez-Mier P, Vinson F, Cooke J, et al. Pathway analysis in metabolomics: Recommendations for the use of over-representation analysis. PLoS Comput Biol. 2021 Sep;17(9):e1009105.
