# Supplementary figures and images for: A strategy to detect metabolic changes induced by exposure to chemicals from large sets of condition-specific metabolic models computed with enumeration techniques
Source: BMC Bioinformatics. 2024 Jul 11;25:234. doi: 10.1186/s12859-024-05845-z (PMC11238488; doi:10.1186/s12859-024-05845-z)

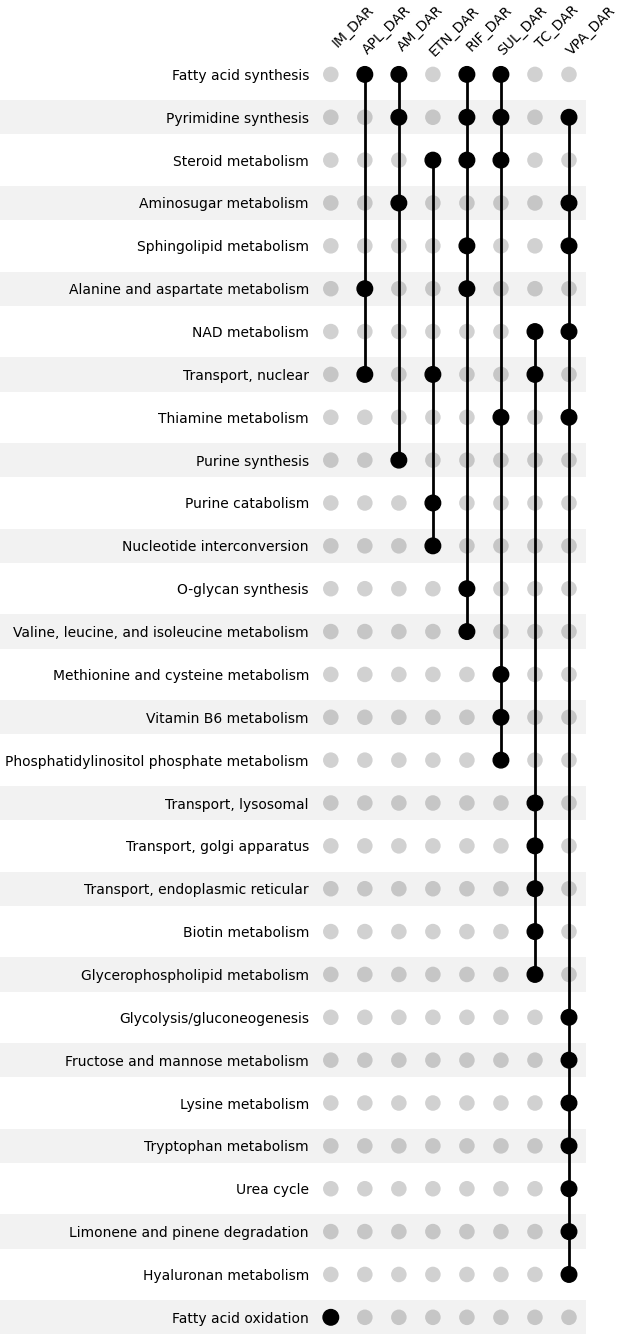

Supplement: Supplementary file 2 — Supplementary Material 2: S1 Fig. Pathway enrichment performed on DARs, after removing blocked and artificial reactions. Pathway over-representation analysis was performed on Recon2.2 metabolic pathways for DARs predicted for the eight selected hepatotoxic molecules? DARs were filtered to remove blocked and artificial reactions. P-values were computed using a Fisher Exact test, with a Benjamini-Hochberg correction. [file 12859_2024_5845_MOESM2_ESM.tiff]

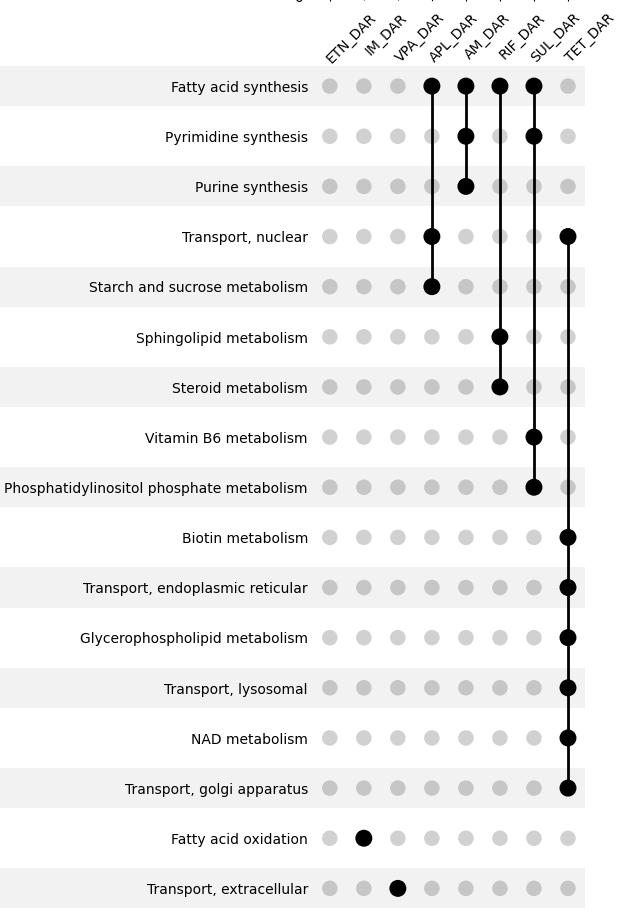

Supplement: Supplementary file 3 — Supplementary Material 3: S2 Fig. Pathway enrichment performed on DARs. Pathway over-representation analysis was performed on Recon2.2 metabolic pathways for DARs predicted for the eight selected hepatotoxic molecules, without removing blocked and artificial reactions. P-values were computed using a Fisher Exact test, with a Benjamini-Hochberg correction. [file 12859_2024_5845_MOESM3_ESM.tiff]

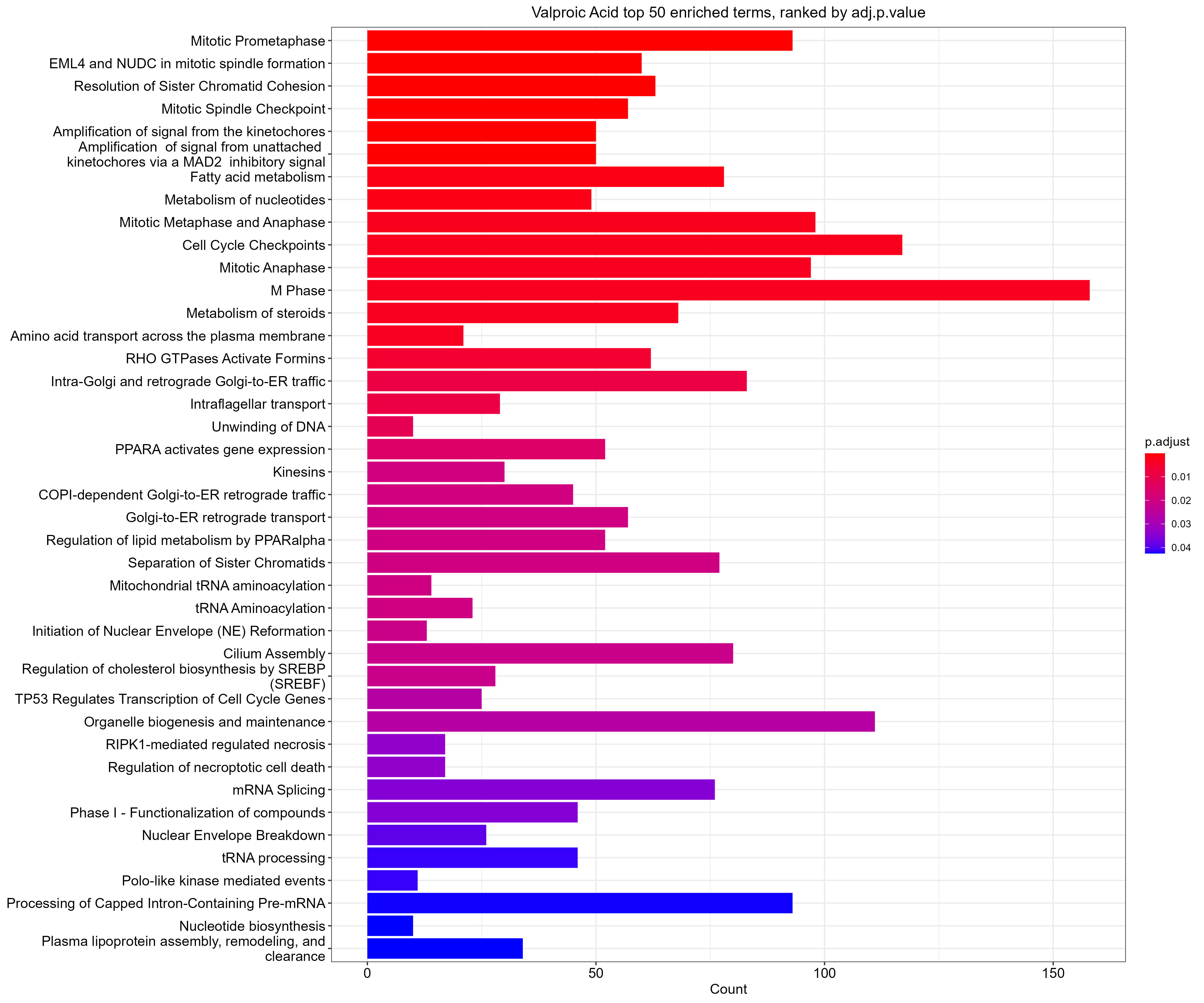

Supplement: Supplementary file 4 — Supplementary Material 4: S3 Fig. Pathway enrichment performed on valproic acid DEGs with ReactomePA. Pathway over-representation analysis performed with a Fisher Exact test, p-values corrected with the Benjamini-Hochberg method on Reactome 2022 pathways (genes with log2(absFC)) > 0.26 and FDR-corrected p-values < 0.05 were considered as DEG). [file 12859_2024_5845_MOESM4_ESM.tiff]

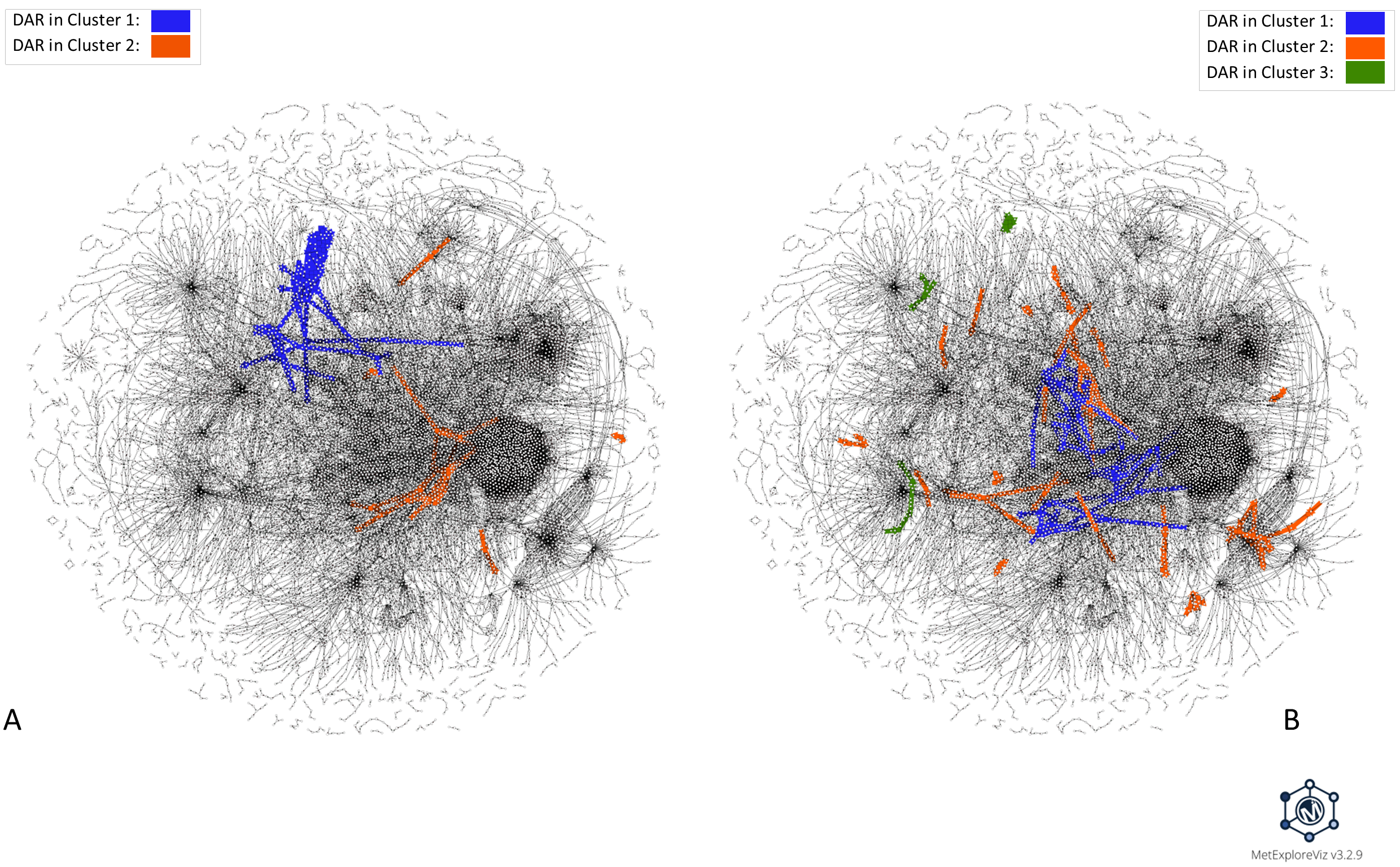

Supplement: Supplementary file 5 — Supplementary Material 5: S4 Fig. Visualization of DARs identified for amiodarone and valproic acid within the Recon2.2 metabolic network, colored by cluster. This visualization was performed with MetExploreViz while removing side compounds (S4 Table). DARs identified for amiodarone (7 µM, 24 h) are highlighted in panel A and DARs identified for valproic acid (5000µM, 24h) are highlighted in panel B. In panel A, DARs highlighted in blue are associated with the cluster 1 for amiodarone and DARs highlighted in orange are associated with the cluster2 for amiodarone. In panel B, DARs highlighted in blue are associated with the cluster 1 for valproic acid, DARs highlighted in orange are associated with the cluster 2 for valproic acid and DARs highlighted in green are associated with the cluster 3 for valproic acid. The two figures are based on the same network layout; thus, each reaction and metabolite is located at the same coordinates, allowing visual comparison. Interactive visualizations can be accessed through these links https://metexplore.toulouse.inrae.fr/userFiles/metExploreViz/index.html?dir=/5b6c886c4916c1de9e6c16a776cc6d64/networkSaved_1726726315 and https://metexplore.toulouse.inrae.fr/userFiles/metExploreViz/index.html?dir=/5b6c886c4916c1de9e6c16a776cc6d64/networkSaved_1522683843. [file 12859_2024_5845_MOESM5_ESM.tiff]
